# Template MRI scans reliably approximate individual and group-level tES and TMS electric fields induced in motor and prefrontal circuits

**DOI:** 10.3389/fncir.2023.1214959

**Published:** 2023-09-06

**Authors:** Jennifer Y. Cho, Sybren Van Hoornweder, Christopher T. Sege, Michael U. Antonucci, Lisa M. McTeague, Kevin A. Caulfield

**Affiliations:** ^1^Department of Neuroscience, Medical University of South Carolina, Charleston, SC, United States; ^2^Faculty of Rehabilitation Sciences, REVAL–Rehabilitation Research Center, Hasselt University, Diepenbeek, Belgium; ^3^Department of Psychiatry, Medical University of South Carolina, Charleston, SC, United States; ^4^Department of Radiology and Radiological Science, Medical University of South Carolina, Charleston, SC, United States; ^5^Ralph H. Johnson VA Medical Center, Charleston, SC, United States

**Keywords:** TMS, tES, tDCS, non-invasive brain stimulation, electric field (E-field) modeling, finite element method (FEM), MNI-152, template MRI scan

## Abstract

**Background:**

Electric field (E-field) modeling is a valuable method of elucidating the cortical target engagement from transcranial magnetic stimulation (TMS) and transcranial electrical stimulation (tES), but it is typically dependent on individual MRI scans. In this study, we systematically tested whether E-field models in template MNI-152 and Ernie scans can reliably approximate group-level E-fields induced in *N* = 195 individuals across 5 diagnoses (healthy, alcohol use disorder, tobacco use disorder, anxiety, depression).

**Methods:**

We computed 788 E-field models using the CHARM–SimNIBS 4.0.0 pipeline with 4 E-field models per participant (motor and prefrontal targets for TMS and tES). We additionally calculated permutation analyses to determine the point of stability of E-fields to assess whether the 152 brains represented in the MNI-152 template is sufficient.

**Results:**

Group-level E-fields did not significantly differ between the individual vs. MNI-152 template and Ernie scans for any stimulation modality or location (*p* > 0.05). However, TMS-induced E-field magnitudes significantly varied by diagnosis; individuals with generalized anxiety had significantly higher prefrontal and motor E-field magnitudes than healthy controls and those with alcohol use disorder and depression (*p* < 0.001). The point of stability for group-level E-field magnitudes ranged from 42 (motor tES) to 52 participants (prefrontal TMS).

**Conclusion:**

MNI-152 and Ernie models reliably estimate group-average TMS and tES-induced E-fields transdiagnostically. The MNI-152 template includes sufficient scans to control for interindividual anatomical differences (i.e., above the point of stability). Taken together, using the MNI-152 and Ernie brains to approximate group-level E-fields is a valid and reliable approach.

## Introduction

Transcranial magnetic stimulation (TMS) and transcranial electrical stimulation (tES) are two methods of non-invasively stimulating the human brain (Barker et al., 1985; Nitsche and Paulus, 2000; Dayan et al., 2013). Using electromagnetic (i.e., TMS) or direct electrical energy (i.e., tES), non-invasive brain stimulation can excite or inhibit the different brain regions via long-term potentiation (LTP) or long-term depression (LTD)-like effects (Chervyakov et al., 2015; Kronberg et al., 2017). Researchers have utilized TMS and tES to stimulate various neural circuits to understand how exciting or inhibiting different brain regions within networks causally affects brain activity (Sack, 2006; Hobot et al., 2021; Grover et al., 2022). In addition, both TMS and tES have been used clinically for treating multiple neurological and psychiatric diagnoses. Most notably, TMS is US FDA-approved to treat depression (O’Reardon et al., 2007; George et al., 2010), depression with anxiety, obsessive compulsive disorder (OCD) (Carmi et al., 2019), migraine headaches, and tobacco use disorder (Zangen et al., 2021). However, while TMS is FDA-approved to treat these four diagnoses, it is not consistently effective for every patient. For instance, once daily TMS for depression has response rates in a naturalistic setting of approximately 50–70% (Carpenter et al., 2012, 2021). While impressive, there is still room for improvement. In addition, tES studies have reported varying results (Horvath et al., 2015), with multiple well-designed clinical trials reporting mixed findings in the treatment of depression (Brunoni et al., 2017; Loo et al., 2018) and in other domains such as working memory (Brunoni and Vanderhasselt, 2014; Papazova et al., 2018; Westwood and Romani, 2018). Thus, there is an ongoing need to further understand and develop more effective TMS and tES treatments for multiple diagnoses. A key consideration to improve TMS and tES efficacy is determining whether a therapeutic level of stimulation engages the cortical target and neural circuit of interest (Zmeykina et al., 2020; Turi et al., 2021). A tool that can elucidate the amount of target and circuit engagement, and potentially improve clinical responses via personalized dosing, is electric field (E-field) modeling.

E-field modeling is a method of accurately estimating how much non-invasive brain stimulation applied at the scalp reaches the cortical level using magnetic resonance imaging (MRI) scans, tissue segmentations/meshing, and tissue conductivity values (Huang et al., 2017; Saturnino et al., 2019). As the magnitude of the E-field affects brain activity in a specific region or network, variability in the induced E-field can subsequently impact clinical response (Suen et al., 2020; Caulfield et al., 2022b; Zhang et al., 2022; Deng et al., 2023). Seminal studies in clinical TMS described how older individuals with larger scalp-to-cortex distances did not respond to treatments suggesting that the induced E-field magnitude is key to maximizing therapeutic response (Kozel et al., 2000; Nahas et al., 2004). Regarding tES, researchers have reported varying effects at differing scalp stimulation intensities in 0.5 mA increments (Moliadze et al., 2012; Batsikadze et al., 2013), highlighting the need for dose standardization and more advanced understanding of tES-induced E-fields at the cortical level. E-field modeling can elucidate dose-response relationships between E-field magnitude and therapeutic response and has broad applications including prospective dosing to ensure patients receive similar E-field intensities at specific brain regions (Caulfield et al., 2020, 2021; Saturnino et al., 2021; Dannhauer et al., 2022). However, most clinical brain stimulation providers do not implement E-field modeling for a variety of reasons, including the difficulty of obtaining structural MRI scans, lack of E-field modeling expertise, and need for advanced equipment such as neuronavigation (Caulfield et al., 2022a).

A possible proxy for using individual MRI scans that has not yet been systematically investigated is whether using a template MRI scan would be a suitable method of approximating group-average E-field values. While a group-level E-field model is incapable of explaining interindividual variability, it is highly informative in the search for what E-field intensities are clinically meaningful, in which populations and setting. As many researchers also retrospectively report the induced E-fields in template or standard brains included in E-field modeling packages (e.g., Konakanchi et al., 2020; Cobb et al., 2021), we sought to assess the accuracy of utilizing template brains to estimate group-level E-field averages. Moreover, utilizing template MRI scans could be useful for exploring general, non-patient specific properties of non-invasive brain stimulation such as the effects of novel tES electrode placements, coil architecture or angle, or estimating group-level effects such as in grant applications or to demonstrate the feasibility of tES or TMS in particular populations.

In this study, our goal was to assess whether using the MNI-152 brain or Ernie brain included in E-field modeling software packages would produce similar E-field values to *N* = 195 TMS participants with T1w MRI scans. If template scan and group-average E-field values were similar, researchers and clinical providers without access to individual MRI scans could still inform and approximate E-field modeling derived values with greater certainty than would be afforded in the absence of computational approaches.

## Materials and methods

### Participants

In total, we included the MRI scans of 195 participants in this E-field modeling study from an initial dataset of *N* = 197; two participants were deemed to have poor segmentation integrity and were excluded from further analysis. Each participant was treated with TMS in six Medical University of South Carolina IRB-approved protocols and provided written informed consent. The 195 participants were comprised of 106 men and 89 women, with an average age = 39.3 ± 14.0 years old and age range = 20–69 years old. Participants had the following five diagnoses: healthy controls (*N* = 31), alcohol use disorder (*N* = 87), tobacco use disorder (*N* = 31), generalized anxiety (*N* = 25), and depression (*N* = 21).

In addition, we utilized the MRI scans of the MNI-152 template brain (Fonov et al., 2011) and Ernie, an MRI scan and head model included in the SimNIBS software package that is commonly used to approximate E-fields. The MNI-152 template is an averaged structural MRI template based on 152 people, including 86 male/66 female brains with an average age = 25.02 ± 4.90 and age range = 18–44 years old. While the demographical information of the Ernie dataset has not been previously published due to PHI considerations, this head model has been used in several E-field modeling studies (e.g., Kalloch et al., 2020; Gomez et al., 2021).

### Motor threshold acquisition procedure

Using a MagVenture MagPro R30 or X100 machine and Cool-B65 TMS coil, TMS operators acquired resting motor threshold (rMT) values for each of the 195 participants using a visual approach. We defined the motor threshold as 5/10 visible anterior pollicis brevis (APB) muscle twitches. The rMT values were an average of 50.46 ± 8.91% (range = 31–78%) of maximal machine output. Therefore, the average 120% rMT stimulation intensity was 60.55 ± 10.70% of maximal machine output. Both TMS machines have a maximal dI/dt stimulator-coil output of 150e6 A/s, ensuring that each participant was stimulated with the same intensity across machines.

As rMTs were not acquired for the MNI-152 and Ernie brains, we simulated TMS using 120% of the group average rMT from the *N* = 195 experimentally determined values to the closest percentage point. This equated to 61% of machine output on the MagVenture MagPro systems with Cool-B65 TMS coil.

### MRI scan parameters

Individual MRI scans were acquired at MUSC on a Siemens PRISMA 3T scanner and 32-channel head coil. Each participant underwent an MPRAGE structural T1w MRI scan with 0.9 mm × 0.9 mm × 0.9 mm isotropic voxels, image size: 256 × 256 × 256 voxels, TR: 2,300 ms, TE: 2.32 ms, TI: 900 ms, acceleration factor PE: 2, 192 slices, fat suppression off. The MNI-152 template brain is an open access composite brain comprised of 152 individuals with 1 mm × 1 mm × 1 mm voxels and image size: 182 × 238 × 282 voxels (Fonov et al., 2011). The Ernie T1w scan was acquired with 1 mm × 1 mm × 1 mm voxels with image size 182 × 238 × 282 voxels.

### E-field modeling overview

**FIGURE 1:**
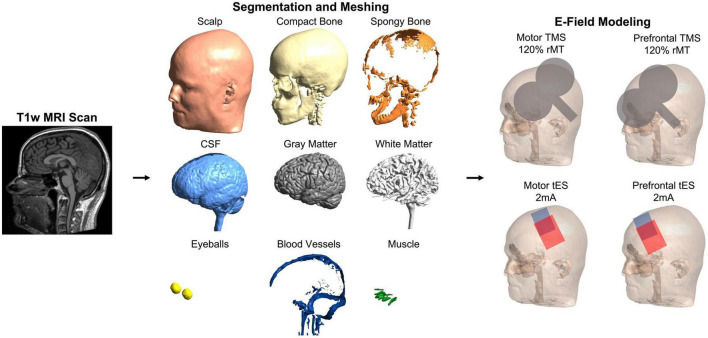
Electric field modeling pipeline. We created 195 individual head models and head models for the MNI-152 template brain and Ernie, an included mesh in the SimNIBS example folder. For each person, we computed four E-field models to simulate the effects of stimulation over the motor and prefrontal cortices with TMS and tES. This figure shows the pipeline in a representative participant.

We utilized SimNIBS 4.0.0 (Saturnino et al., 2019) and the CHARM segmentation and meshing pipeline (Puonti et al., 2020) for E-field modeling based on individual MRI scans acquired in each participant and the MNI-152 and Ernie scans (Figure 1). In total, we created *N* = 195 individual head models and one head model each for the MNI-152 brain and the Ernie brain. CHARM segments and meshes MRI scans into anatomically accurate tetrahedral head models comprised of 9 tissue layers: scalp, compact bone, spongy bone, cerebrospinal fluid (CSF), gray matter, white matter, eyeballs, blood vessels, and muscle. We assigned standard tissue conductivity values (Wagner et al., 2004; Opitz et al., 2015; Saturnino et al., 2015) to each tissue type (Scalp: 0.465 S/m, compact bone: 0.008 S/m, spongy bone: 0.025 S/m, CSF: 1.654 S/m, gray matter: 0.275 S/m, white matter: 0.126 S/m, eyeballs: 0.5 S/m, blood vessels: 0.6 S/m, and muscle: 0.16 S/m). As each tissue is assigned a different conductivity value, E-field modeling can accurately estimate the magnitude of stimulation that reaches the cortex in both TMS and tES. We visually inspected each head model to ensure the accuracy of segmentation and meshing. Due to this process, the head models for two individuals from an initial 197 scans were excluded due to noticeable intersections between tissue layers.

### TMS E-field modeling

**FIGURE 2:**
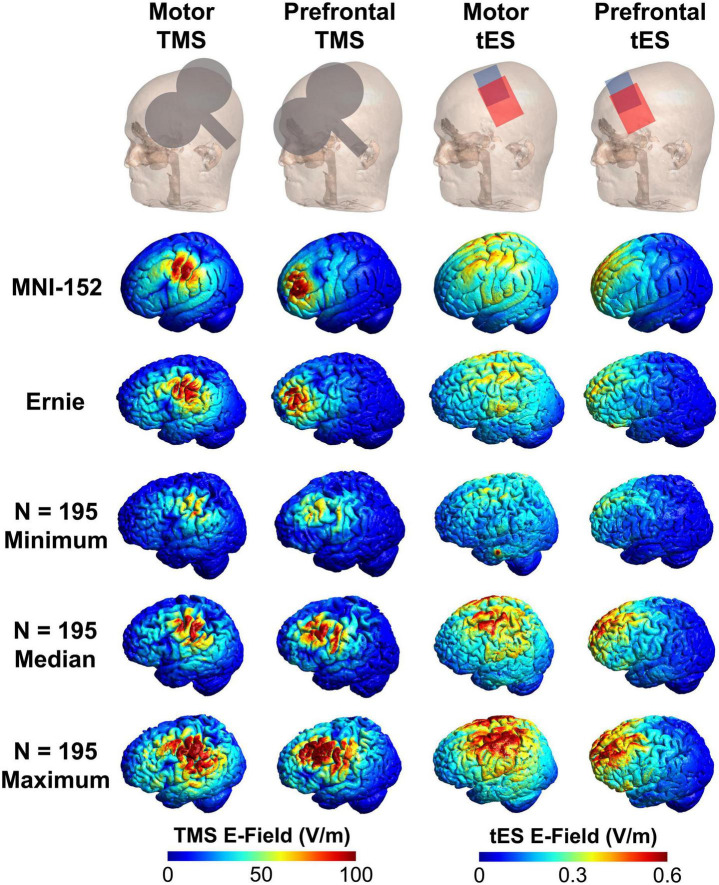
Visual comparison of the MNI-152, Ernie, and *N* = 195 individual E-field models. Here, we show the TMS coil and tES electrode placements, and E-field models in the MNI-152 and Ernie brains, as well as the E-field models in the *N* = 195 participants with the minimum, median, and maximum E-fields. Qualitatively, the MNI-152 and Ernie brains produce similar E-field magnitudes as the median models for each of the four stimulation types.

Using the 10-10_UI_Jurak_2007 EEG coordinate file output in SimNIBS (Jurcak et al., 2007), we centered the simulated MagVenture_Cool-B65 coil model over C3 (motor) and F3 (prefrontal) (Beam et al., 2009) stimulation targets in two E-field models (Figures 1, 2). For stimulation intensity, we calculated the dI/dt value in A/s, with a maximum stimulator-coil output of 150e6 A/s. Using the motor threshold values, we calculated the 120% motor threshold stimulator output and multiplied this value by the maximum stimulator output (e.g., 50% stimulator output = 75e6 A/s). Custom MATLAB scripts ensured that the coil angle was oriented exactly 45° relative to the sagittal plane in each model.

### tES E-field modeling

For tES simulations, we bilaterally centered electrodes over the motor (C3 and C4) or prefrontal (F3 and F4) cortices using the same 10-10_UI_Jurak_2007 EEG coordinate file (Figures 1, 2). We used conventional 7 cm × 5 cm pad electrodes with the longer electrode axis oriented left/right on each participant’s scalp. tES was simulated using 2 mA electrical current with the anodal electrode placed over the left hemisphere (C3 and F3) and the cathodal electrode placed over the right hemisphere (C4 and F4). While there are other commonly used electrode placements such as C3-supraorbital (SO) and F3-SO, we chose bilateral placements as they are commonly used and previous reports have found no significant differences in large-scale modeling between bilateral C3-C4 and C3-SO placements with 7 cm × 5 cm pad electrodes (Caulfield and George, 2022).

### E-field magnitude outcome measure

To examine the E-field at the cortical target, we utilized two region of interest (ROI) analyses for both the TMS and tES models. Both ROIs extracted the average E-field within a spherical volume with a radius of 10 mm with a gray matter mask, as has been previously reported in the literature (e.g., Caulfield et al., 2022a; Caulfield and George, 2022). At a post-processing step, the CHARM segmentation pipeline fits the MNI template brain to the individual scan enabling researchers to perform analyses using standardized MNI coordinates across participants. Notably, this MNI template fitting does not affect the segmentation and meshing process as CHARM was trained on *N* = 20 individual head models.

For each person, the ROIs were centered over the stimulation target in the left hemisphere at motor MNI coordinate: −52.2, −16.4, 57.8 and prefrontal MNI coordinate at −35.5, 49.4, 32.4, based on publications reporting the locations of the cortical projections from scalp locations C3 (motor cortex) and F3 (prefrontal cortex) (Okamoto et al., 2004; Okamoto and Dan, 2005).

### Statistical measures

We conducted two types of statistical analyses to assess the suitability of utilizing the MNI-152 and Ernie scans and head models in lieu of having individual MRI scans. First, we used four one-way ANOVAs to assess the group-level differences between individual MRI scan E-field models and the MNI-152 and Ernie E-field models (four ANOVAs; one ANOVA each for motor TMS, prefrontal TMS, motor tES, and prefrontal tES). In addition, we computed additional one-way ANOVAs measuring the effects of diagnosis or sex on E-field results and differences between individual, MNI-152, and Ernie scans (eight total ANOVAs; one set of four ANOVAs for diagnosis and one set of four ANOVAs for sex). For sex, we accounted for conditions of men in the *N* = 195 sample, women in the *N* = 195 sample, the MNI-152 template, and Ernie. We chose to not examine the effects age on E-field magnitudes due to the collinearity of age and increased scalp-to-cortex distances already inherently being accounted for in E-field models and the unknown age of Ernie. All ANOVAs were calculated using SPSS 27.0 (IBM Corp., Armonk, NY, USA). For all statistical measures, the significance level was set to α = 0.05 (two-tailed).

Second, to determine the minimum number of E-field models on individual scans to obtain a stable E-field value, we performed four permutation statistical analyses on the 195 individual E-field models in MATLAB R2022b (The Mathworks Inc., Natick, MA, USA). Our approach was based on the work of Van Hoornweder et al. (2022a). In the sample of *N* = 195 E-field models for each stimulation paradigm (i.e., motor TMS, prefrontal TMS, motor tES, and prefrontal tES), we randomly selected subsamples with increasing size from *N* = 1 to *N* = 195, repeating the procedure 10,000 times per subsample (i.e., 1,950,000 subsamples for each permutation). We chose to combine the heterogenous populations to maximize the likelihood of determining a corridor of stability since each individual diagnostic population had only *N* = 21 to *N* = 87 participants. We defined a corridor of stability between the 5 and 95th percentile range of the entire sample. In line with prior methodology, we calculated the “point of stability” which we defined as the point where the mean of the randomly selected subsample enters the corridor of stability and does not leave it at increasing subsample sizes. Our primary goal in determining the point of stability was to assess how many individual scans and subsequent E-field models would be needed to produce stable E-field values on the group level; if this number were larger than 152, it would suggest that a template brain with more scans than the MNI-152 composite scan would be necessary to accurately estimate group-level E-field models. All data are reported as mean ± standard deviation (SD).

## Results

### TMS electric field magnitudes

**FIGURE 3:**
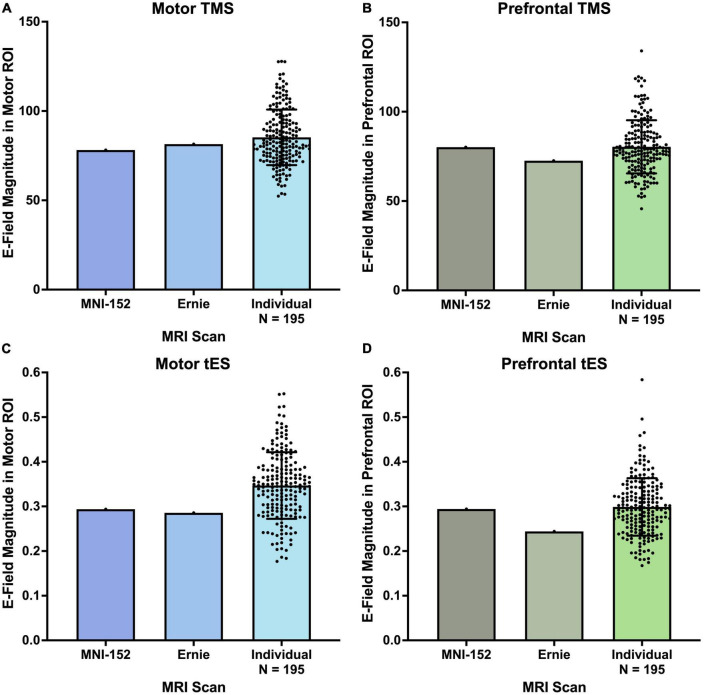
E-field magnitudes produced from the MNI-152, Ernie, and *N* = 195 individual head models. There were no significant differences between the E-field magnitudes produced at motor and prefrontal ROIs between the MNI-152, Ernie, and *N* = 195 individual models, suggesting that template brains can reliably approximate the E-fields produced on a group level. Error bars denote ± standard deviation.

For both motor and prefrontal TMS-induced E-fields, the 195 individual MRI scans did not significantly differ from the MNI-152 and Ernie brains (Figures 3A, B). TMS-induced motor E-fields did not significantly differ amongst the individual brains (85.3 ± 15.5 V/m), MNI-152 template (78.2 V/m), and Ernie brain (81.5 V/m), F(2, 194) = 0.13, *p* = 0.88, η_*p*_^2^ = 0.001 (Figure 3A and Table 1). Similarly, TMS-induced prefrontal E-fields did not significantly differ between individual brains (80.3 ± 14.9 V/m), the MNI-152 template (80.1 V/m), and Ernie brain (72.6 V/m), F(2, 194) = 0.14, *p* = 0.87, η_*p*_^2^ = 0.001 (Figure 3B).

**TABLE 1:** Electric field means and standard deviations (in parentheses) of MNI-152, Ernie, transdiagnostic, and individual diagnostic populations.

	Motor TMS (V/m)	Prefrontal TMS (V/m)	Motor tES (V/m)	Prefrontal tES (V/m)
MNI-152	81.46	80.14	0.293	0.294
Ernie	78.16	72.58	0.286	0.244
Transdiagnostic (*N* = 195)	85.26 (15.54)	80.35 (14.88)	0.347 (0.075)	0.299 (0.064)
Anxiety (*N* = 25)	95.76 (15.90)	88.75 (13.44)	0.350 (0.082)	0.289 (0.062)
Tobacco use disorder (*N* = 31)	90.96 (15.52)	87.30 (17.74)	0.376 (0.098)	0.318 (0.087)
Healthy controls (*N* = 31)	84.20 (14.48)	79.23 (13.11)	0.343 (0.059)	0.310 (0.052)
Alcohol use disorder (*N* = 87)	81.60 (13.66)	76.35 (12.43)	0.341 (0.064)	0.291 (0.057)
Depression (*N* = 21)	81.11 (17.17)	78.30 (17.25)	0.327 (0.080)	0.299 (0.070)

We additionally measured the differences in TMS-induced E-fields by diagnosis, finding that there were significant effects of diagnosis on E-field magnitude in both motor [F(4, 190) = 6.09, *p* < 0.001, η_*p*_^2^ = 0.114] and prefrontal TMS [F(4, 190) = 5.96, *p* < 0.001, η_*p*_^2^ = 0.111]. See Table 1 for detailed E-field magnitudes reported by diagnosis. For motor TMS, *post hoc* Tukey-corrected analyses revealed significant differences between generalized anxiety (95.8 ± 15.9 V/m) compared to healthy controls (84.2 ± 14.5 V/m), alcohol use disorder (81.6 ± 13.7 V/m), and depression (81.1 ± 17.2 V/m). We found additional differences between the motor E-field magnitudes produced in tobacco use disorder (91.0 ± 15.5 V/m) and alcohol use disorder. For prefrontal TMS, participants with generalized anxiety (88.8 ± 13.4 V/m) and tobacco use disorder (87.3 ± 17.7 V/m) had significantly higher E-fields than those with alcohol use disorder (76.3 ± 12.4 V/m).

Regarding the effects of sex on TMS-induced E-fields, we found no significant differences between E-field magnitudes for men, women, the MNI-152, or Ernie brains. For motor TMS models, women (87.7 ± 15.5 V/m), men (83.2 ± 15.4 V/m), the MNI-152 template (78.2 V/m), and Ernie (81.5 V/m) did not significantly differ in E-field magnitude, F(3, 193) = 0.89, *p* = 0.45, η_*p*_^2^ = 0.014, F(3, 193) = 1.42, *p* = 0.24, η_*p*_^2^ = 0.022. Similarly, for prefrontal TMS models did not significantly differ between populations of women (82.1 ± 14.8 V/m), men (78.8 ± 14.8 V/m), the MNI-152 template (80.1 V/m), and Ernie (72.6 V/m), F(3, 193) = 0.89, *p* = 0.45, η_*p*_^2^ = 0.014.

### tES electric field magnitudes

Likewise, both the motor and prefrontal tES E-fields were similar between the *N* = 195 individual scans, the MNI-152 template, and Ernie brains (Figures 3C, D). Motor tES E-fields did not significantly differ between the individual brains (0.35 ± 0.075 V/m), MNI-152 template (0.29 V/m), and Ernie brain (0.29 V/m), F(2, 194) = 0.58, *p* = 0.56, η_*p*_^2^ = 0.006 (Figure 3C). Furthermore, prefrontal tES E-fields did not significantly vary between individual brains (0.30 ± 0.06 V/m), the MNI-152 template (0.29 V/m), and Ernie brain (0.24 V/m), F(2, 194) = 0.36, *p* = 0.70, η_*p*_^2^ = 0.004 (Figure 3D).

In contrast to the TMS-induced E-fields, tES E-fields did not differ by diagnosis for both motor [F(4, 190) = 1.76, *p* = 0.14, η_*p*_^2^ = 0.036] and prefrontal tES [F(4, 190) = 1.42, *p* = 0.23, η_*p*_^2^ = 0.029]. See Table 1 for specific E-field magnitudes by diagnosis.

With regards to the effects of sex on tES-induced E-fields, we found no significant differences between E-field magnitudes for men, women, the MNI-152, or Ernie brains. For motor tES, there were no significant differences between women (0.36 ± 0.082 V/m), men (0.33 ± 0.066 V/m), the MNI-152 template (0.29 V/m), and Ernie brains (0.29 V/m), F(3, 193) = 2.52, *p* = 0.06, η_*p*_^2^ = 0.038. Likewise, for prefrontal tES, there were no significant differences between women (0.30 ± 0.069 V/m), men (0.30 ± 0.061 V/m), MNI-152 template (0.29 V/m), and Ernie brains (0.24 V/m), F(3, 193) = 0.42, *p* = 0.74, η_*p*_^2^ = 0.006.

### Sample size stability analyses

**FIGURE 4:**
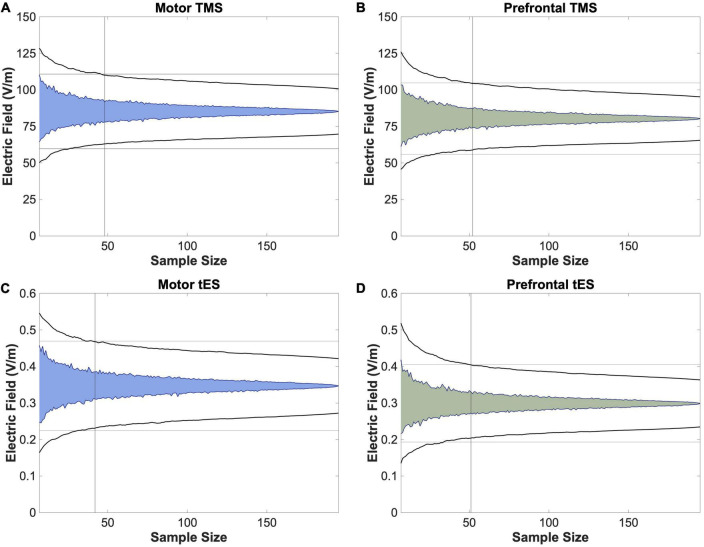
Permutation analyses to determine stable sample sizes in *N* = 195 participants. To further analyze whether utilizing a template scan is an appropriate method of approximating group-level E-field magnitudes of different stimulation paradigms, we performed bootstrapping analyses to determine the stable sample size at which the E-fields produced remained within the 95% confidence interval, as denoted with a vertical line. We found that this number ranged from 42 (motor tES) to 52 (prefrontal TMS). As 52 participants is lower than the 152 scans included in the MNI-152 template, these data suggest that utilizing template scans may be a method of estimating stable group-level E-field magnitudes.

Using four permutation analyses, we determined the minimum number of participants and E-field models needed to produce stable group-level E-field values in this heterogenous *N* = 195 group (Figure 4). For motor TMS, the stable E-field value was achieved at *N* = 48 participants compared to *N* = 52 for prefrontal TMS (Figures 4A, B). Regarding motor tES, *N* = 42 participants were needed to achieve stability, versus *N* = 51 for prefrontal tES (Figures 4C, D).

## Discussion

In this study, we assessed the utility of calculating TMS and tES E-field models in the template MNI-152 scan and Ernie brain compared to *N* = 195 participants with 5 diagnoses (i.e., healthy controls, alcohol use disorder, tobacco use disorder, generalized anxiety, and depression). We simulated four common non-invasive brain stimulation protocols (i.e., motor and prefrontal TMS and tES) per participant for 788 total E-field models. We found that there were no significant group-level differences of the E-field magnitudes induced from motor and prefrontal TMS and tES from individual scans vs. MNI-152 and Ernie brains. For TMS, the MNI-152 template produced 8.3 and 0.3% lower E-fields for the motor and prefrontal cortices, respectively, while the Ernie brain had 4.5 and 9.7% lower E-fields than the group average motor and prefrontal E-fields from the *N* = 195 participants (Figure 3 and Table 1). There were more pronounced, albeit still non-significant differences for tES, with the MNI-152 template producing 16.3 and 1.6% lower E-fields and the Ernie brain having 17.7 and 18.4% lower E-fields in the motor and prefrontal cortices, respectively, than the *N* = 195 individual models. Thus, while there were no significant overall E-field magnitude differences, it appears that the MNI-152 and Ernie brains are most accurate at estimating the group-level regions and neural circuits simulated by TMS, compared to tES. In conjunction with prior reports (Minjoli et al., 2017; Tzirini et al., 2022), MNI-152 and Ernie brains may be most accurate at estimating TMS-induced E-fields due to TMS being less affected by individual tissue composition due to the electromagnetic stimulation approach compared to the direct electrical stimulation method utilized in tES, which is more heavily governed by the underlying tissue composition.

It is also important to consider the effects of diagnosis on E-field magnitude. Here, we reported that TMS-induced E-fields differ as a product of diagnosis, such that individuals with generalized anxiety and tobacco use disorder had significantly higher motor and prefrontal E-fields than those with alcohol use disorder. In addition, individuals with generalized anxiety had significantly higher motor E-fields than healthy controls and people with depression. Interestingly, these nuanced relationships between E-field magnitude and diagnosis only existed for TMS and not tES, as all tES-induced E-fields did not differ by diagnosis (Table 1). This may be in part due to uniform tES applying the same stimulation intensity of 2 mA across models whereas our TMS models used the experimentally-determined dI/dt values based on individual motor thresholds determined for each person. Since a uniform stimulation intensity was applied across each person, fixed dose 2 mA tES may have reduced the amount of variation between individuals that individualized motor threshold values provide. These data point at the utility of personalized dosing for tES as certain diagnoses likely require a higher individualized dosage for appropriate target engagement of cortical targets and neural circuits. Moreover, since we found no group-level differences combining across diagnoses between *N* = 195 TMS and tES E-fields and template MNI-152 and Ernie brains, this relationship may change depending on the different diagnoses considered. Therefore, future research should consider further investigating the appropriateness of using template MNI-152 and Ernie brains to estimate group-level E-fields in different populations. Likely, these TMS-induced E-field data indicate differing neurophysiology and the up- or down-regulation of neural circuits across diagnostic populations. In comparison, similar tES-induced E-fields across diagnoses might indicate that the scalp-to-cortex distance and tissue compositions may be relatively similar across populations, as reflected by the similar tES-induced E-field magnitude values. Future research could further elucidate the modality-specific findings and interactions with diagnoses that we reported here.

In a second series of analyses, we used a permutation approach to compute the point of stability at which the group average E-field value did not increase in variance with additional E-field models (i.e., the point of stability). The point of stability differed slightly by stimulation modality and location, with a range of 42 to 52. Notably, the point of stability across stimulation modalities was always well short of the 152 scans included in the MNI-152 brain, further validating the composite brain as having a high enough number of scans that the interindividual variation is likely appropriately represented. Taken together, these data validate the strategy of using a template MNI-152 brain scan to approximate group-level E-field results as it takes many scans into consideration that average across neuroanatomical idiosyncrasies.

There are numerous implications of this research and how template MRI scans could be utilized to estimate how much stimulation reaches the cortex. First, while the utility of using template brains for E-field modeling is question-dependent, it appears that template brains can reliably estimate group-level effects, even in a clinically heterogenous group. We report that the group-level E-field values from individual MRI scans do not significantly differ from the E-field values produced from MNI-152 and Ernie brains, validating the use of template scans to estimate group-level E-field values. Using template scans for E-field modeling could have multiple uses. For instance, using template scans for E-field modeling could inform the effects of more sophisticated and optimized electrode positioning, sizes, and inter-electrode distances such as through high definition tES (HD-tES) or anterior posterior pad surround tES (APPS-tES), as has been done in prior publications (Datta et al., 2009; Deng et al., 2013; Saturnino et al., 2015; Laakso et al., 2016; Caulfield and George, 2022). As many tES studies do not acquire MRI scans, particularly with the increasing use of at-home tES (André et al., 2016; Riggs et al., 2018; Pilloni et al., 2022), using a template MRI scan to plan more optimized tES electrode positioning and intensity for a specific goal may help to ensure that a therapeutic cortical intensity is induced at the right target on the group level.

It is also interesting to compare our findings to prior efforts looking at datasets of 60 or more individual E-field models such as the study by Laakso et al. (2016). Similar to our results, these researchers reported more variable frontal tES-induced E-fields than motor E-fields, substantiating our finding that prefrontal tES-induced E-fields required a greater number of participants to obtain a point of stability (Figure 4). This is notable as Laakso et al. (2016) included 64 healthy younger adults whereas we collapsed across 5 transdiagnostic groups, suggesting that our results might hold true in individual populations. It is also important to note other potential differences between our study and the one by Laakso et al. (2016), including different E-field measures (ROI magnitude vs. whole brain normal component). Further research with larger sample sizes per diagnostic condition is needed.

Furthermore, tES has widespread clinical potential but there have been mixed results to date, possibly in part to different amounts of stimulation reaching the cortex with uniform 2 mA dosing. Personalized E-field dosing is an approach that could standardize the stimulation intensity at the cortical target based on varying the dose at the scalp (Caulfield et al., 2020). While personalized E-field dosing is not possible using a template scan, our hope is that we have validated the approach of modeling on the MNI-152 or Ernie brains to provide reliable general estimations for how much stimulation is reaching the cortex in an average person. In turn, the information obtained from quantifying how much stimulation is reaching the cortex on average will surely benefit the ongoing search for the optimal E-field magnitude to maximize non-invasive brain stimulation effects within specific clinical populations and settings (Wischnewski et al., 2021; Alekseichuk et al., 2022; Caulfield et al., 2022b).

Moreover, it is now a common practice to include E-field models in brain stimulation publications and grant applications to substantiate experimental choices of where to position the TMS coil or tES electrodes and which stimulation intensity to choose. As such, including E-field models are informative and allow peers to assess whether an appropriate amount of stimulation reaches the cortex and adequately stimulates the neural circuit of interest. In lieu of having already acquired MRI scans, many researchers perform these E-field models on the MNI-152 template brain (Konakanchi et al., 2020; Cobb et al., 2021), or on the Ernie brain (Kalloch et al., 2020; Gomez et al., 2021) included in the SimNIBS software package. Our study validates the strategy of performing E-field modeling on these MNI-152 and Ernie head models as they can reliably approximate group-level E-field effects of how much and where in different brain regions and neural circuits the stimulation reaches the cortex. Furthermore, it was previously unclear whether the use of the MNI-152 template brain, comprised of healthy adults, would adequately estimate the stimulation intensity in clinical populations. While our scope was limited to diagnoses of mental health and healthy participants, our finding that the group-level E-fields induced from motor and prefrontal tES and TMS did not differ from the template brain suggests that template brain models produce roughly the same E-field magnitudes transdiagnostically. This work should be further evaluated in other diagnoses as particular populations (e.g., stroke) could have greater E-field differences compared to template scan E-fields, especially based on lesion location (Minjoli et al., 2017; Mantell et al., 2021).

While the specific research question dictates the most suitable E-field modeling approach, these data suggest an upper sample size limit of what researchers might consider for future group-level E-field modeling studies. Since it is relatively simple to scale up the number of models and participants in modeling approaches, some researchers have included hundreds of participants with the goal of obtaining stable E-field values that were not significantly impacted by outliers (e.g., Caulfield and George, 2022). Our bootstrapping data suggest that for research questions about the group-level effects of E-field modeling, there is no additional increase in the variation of modeling results above 52 individuals for any stimulation modality. Thus, if the experimenter includes more participants with the objective of reducing the variation of group-level E-field estimates, there are diminishing returns above the point of stability. However, it is important to note that prior findings suggesting that the point of stability is higher than *N* = 52 were likely biased by larger sample sizes (Van Hoornweder et al., 2022a), as is the case in any permutation approach. Future research might also consider performing permutation analyses that separate participants by diagnosis to investigate whether there are differences in the point of stability by condition.

Finally, while we primarily considered how E-field modeling in a template brain might be able to inform group-level analyses, it is important to substantiate how an individually selected E-field value would compare to the MNI template brain estimate (see Figure 2 for minimum, median, and maximum E-fields in the *N* = 195 sample compared to the MNI-152 template and Ernie brain). We reported the following averages and ranges of E-field magnitudes produced from TMS and tES: motor TMS: 85.3 ± 15.5 V/m; prefrontal TMS: 80.3 ± 14.9 V/m; motor tES: 0.35 ± 0.07 V/m; prefrontal tES: 0.30 ± 15.5 V/m. While examining individual E-fields in retrospective or prospective study designs necessitates having individual MRI scans, considering these averages ± 2 SD provides a 95% confidence interval for E-field magnitudes in these TMS and tES protocols. Using these ranges, there were between 7 and 10 individuals falling outside of 2 SD in each stimulation protocol. That the MNI-152 template and Ernie head models produced similar E-field values as the group average values suggests that they are suitable proxy head models for estimating group-average E-fields and that most people (i.e., approximately 95% of people) do not significantly differ on an individual-by-individual basis from the E-fields produced in the MNI-152 brain.

### Limitations and future directions

Briefly, it is important to consider the limitations of this study. We used the MNI-152 brain as a commonly utilized template, but there may be closer matching composite MRI scans depending on the population of interest. For instance, if a researcher wanted to investigate the group-level E-fields in an aging population could consider using more specific age-matched (Fillmore et al., 2015) or diagnosis-matched (Dadar et al., 2022) templates for potentially more accurate simulations. While we used bilateral electrode placements (i.e., C3-C4 and F3-F4) instead of other common electrode placements (e.g., C3-SO and F3-SO), prior large-scale findings have shown that there are no significant differences in E-field magnitudes between C3-C4 and C3-SO (Caulfield and George, 2022). This prior results leads us to believe that the MNI template and Ernie scan would be similarly suitable to accurately estimate E-field magnitudes at other similar electrode placements. In addition, we only utilized a T1w MRI scan for E-field modeling in our naturalistic sample, based on the MRI scans acquired across the parent studies. However, prior reports have described how the inclusion of both T1w and T2w MRI scans improves segmentation accuracy at the skull-CSF border (Nielsen et al., 2018; Van Hoornweder et al., 2022b) and how this can affect E-field modeling values in a regionally-specific fashion (Van Hoornweder et al., 2022). Furthermore, we chose a spherical ROI as our outcome measure, but this may not have encapsulated the peak E-fields induced from tES. Prior results have reported that the maximal E-field is not always located underneath the center of tES electrodes (Caulfield and George, 2022) in the conventional bilateral electrode placement. Forthcoming research has also highlighted the importance of considering the focality of stimulation and recommended reporting both ROI and percentile-based outcome measures (Van Hoornweder et al., 2023), which we did not do here. Thus, extending these data to report percentile-based E-fields could more broadly inform future studies and grant applications utilizing the template scan approach. With larger sample sizes, future analyses could extend the point of stability analyses to specific diagnoses (e.g., healthy controls or participants with anxiety). This method could help to inform the needed number of participants to achieve stable E-field values in these diagnoses as opposed to the transdiagnostic approach that we took here. Finally, we focused on superficial brain targets in this study due to these regions being the primary targets in TMS and tES. In addition, it is more difficult to standardize the tissue compositions between participants at deeper targets due to varying levels of gray vs. white matter. Future research could consider examining deeper targets in more depth, but this was beyond the scope of the current study.

## Conclusion

Utilizing the MNI-152 template scan and Ernie brain produce similar group-level estimations of TMS and tES-induced E-field magnitudes over the motor and prefrontal cortices. Using the MNI-152 brain to approximate group-level E-field effects can provide valuable insight into the amount of stimulation reaching different cortical regions and neural circuits in lieu of individual MRI scans. While preliminary, TMS-induced E-field magnitudes, but not tES-induced E-field magnitudes differed in some diagnoses, including higher motor and prefrontal E-fields in participants with generalized anxiety than alcohol use disorder, healthy controls, and those with depression. Further research is needed to further elucidate the relationships between different diagnoses and E-fields, and whether these impact response to TMS and tES treatments.

## Data availability statement

The datasets presented in this article are not readily available because these data are part of ongoing studies but will be made available upon reasonable request. Requests to access the datasets should be directed to KC, caulfiel@musc.edu.

## Ethics statement

The studies involving humans were approved by Medical University of South Carolina IRB. The studies were conducted in accordance with the local legislation and institutional requirements. The participants provided their written informed consent to participate in this study.

## Author contributions

All authors significantly contributed to the study conceptualization, data analysis, figures creation, writing the original document, editing the document prior to submission, and approved the submitted version.
